# Early Neurodevelopmental Milestones in Children With Autism Spectrum Disorder: A Retrospective Observational Study

**DOI:** 10.7759/cureus.99042

**Published:** 2025-12-12

**Authors:** Dora Sousa, Joana C Queirós, Teresa Tavares, Sara Soares, Inês Vaz Matos, Catarina Prior, Diana Gonzaga

**Affiliations:** 1 Pediatrics, Centro Materno Infantil do Norte Albino Aroso, Centro Hospitalar Universitário do Porto, Porto, PRT; 2 Neurodevelopment Unit, Centro Materno Infantil do Norte Albino Aroso, Centro Hospitalar Universitário do Porto, Porto, PRT

**Keywords:** autism, developmental delay, neurodevelopment, pediatric, risk factors

## Abstract

Introduction

Early diagnosis and intervention in autism spectrum disorder (ASD) are crucial for improving cognitive, social, and adaptive outcomes. ASD is characterized by deficits in social communication and interaction, alongside restricted and repetitive behaviors. Although diagnosis typically occurs around age three, developmental concerns often emerge within the first year, particularly in language, social engagement, and adaptive functioning. Early identification of these markers allows timely referral, assessment, and intervention. This study aimed to characterize early neurodevelopmental milestones in preschool children with ASD, compare milestone acquisition with normative expectations using Haizea-Llevant charts, and evaluate associations with cognitive and adaptive functioning using the Griffiths Mental Development Scales-Extended Revised (GMDS-ER).

Materials and methods

A retrospective observational study included 127 children with ASD followed at a tertiary hospital Neurodevelopment Unit. Data from medical records included demographics, prenatal and family history, comorbidities, age at referral and diagnosis, and early clinical signs. Six developmental milestones were analyzed: social smile, sitting without support, walking, first words, first phrases, and urinary daytime sphincter control. Delays were defined as acquisition at or above the 95th percentile of the Haizea-Llevant chart or absence of the milestone. Language regression, defined as loss of at least five previously acquired words for ≥3 months, was recorded. Cognitive and adaptive functioning were assessed using GMDS-ER, including global development quotient (GDQ) and subscales for personal/social, hearing/language, locomotor, hand-eye coordination, and performance. Statistical comparisons between normative and delayed milestone acquisition were performed.

Results

Of the 127 children, 85.8% were male, with a mean age at diagnosis of 34±10 months. Language delay was the most frequent reason for referral; 11% had experienced regression. Social smile was delayed in 20.5%, sitting in 2.5%, walking in 38.6%, first words in 62.6%, first phrases in 96.8%, and daytime sphincter control in 85.2%. GMDS-ER assessments in 62.2% of children showed a mean GDQ of 61.8±12.7. Delayed sphincter control was associated with lower GDQ (60.2 vs. 76.2, p=0.002), personal/social (56.7 vs. 70, p<0.01), hearing/language (42 vs. 51.7, p=0.048), and performance scores (69.3 vs. 78, p=0.011). Delayed social smile correlated with lower personal/social scores (51.1 vs. 60, p=0.03). Delays in first words (56.7 vs. 64, p=0.014) and phrases (58 vs. 80.5, p=0.023) were linked to reduced personal/social outcomes. Delayed walking was associated with lower performance scores (62.8 vs. 72.8, p=0.025).

Discussion and conclusion

Motor milestones in the first year, except walking, were generally achieved on time. Delayed walking, language acquisition, and daytime sphincter control were strongly associated with lower cognitive, social, and adaptive outcomes. Delayed social smile was associated with poorer personal/social functioning. Careful monitoring of early milestones, particularly social, language, and adaptive skills, can facilitate early ASD identification and guide timely, individualized interventions that may improve long-term outcomes.

## Introduction

Autism spectrum disorder (ASD) or autism is a complex neurodevelopmental condition that often manifests early in childhood and is characterized by deficits in communication and social interaction, and restricted and repetitive behaviors and interests [1]. ASD is a lifelong condition, and a reliable diagnosis is usually not officially made before the age of three years [2]. Autism is considered a descriptive diagnosis, meaning it describes the observed characteristics and symptoms without necessarily indicating a specific underlying cause or pathology [3]. The term spectrum underlines the dimensional nature of the characteristics of the disorder as well as the differences in severity and in the presentation of neurodevelopmental symptoms [2].

The global prevalence of ASD is approximately 1-2% in the general population, witnessing a significant rise in diagnosis in recent decades [2]. The average male-to-female ratio is around 3-4:1. Research has demonstrated that both advanced maternal and paternal age are associated with an elevated risk of having a child with ASD. Risk factors for autism include older parental age, birth trauma, especially if it involves indicators of oxygen deprivation, maternal obesity, a short time between pregnancies, gestational diabetes, and the use of valproic acid during pregnancy [2-4]. ASD frequently occurs alongside other neurodevelopmental disorders, and around 31% of children with ASD also have comorbid intellectual disability [5,6]. There is a broad range of variation within the spectrum, where individuals can exhibit average intelligence or experience varying degrees of intellectual disability [7].

According to a 2010 comprehensive surveillance study conducted on a sample of more than 2000 children diagnosed with ASD, it was found that 83% of them had an additional neurodevelopmental diagnosis [8]. Additionally, 10% of the children had at least one psychiatric diagnosis, and 16% had at least one neurologic diagnosis. In 2020, the American Psychiatric Association documents that more than 95% of people with autism had at least one neurologic or psychiatric additional disorder, many of them with multiple co-occurring problems [2,8].

The Ruth Griffiths Mental Development Scales is a useful tool in the formal assessment of several domains of neurodevelopment. It can be used from infancy to the age of eight years, determining the age at which a typically developing child would accomplish specific tasks and highlighting areas within domains that have not been achieved [9,10]. As a customary component of pediatric healthcare, the assessment of specific developmental milestones in young children, along with developmental screening, plays a crucial role in the early identification of any potential delay, thereby enabling timely intervention. The process starts with screening the general pediatric population to identify children at risk of developmental delay, followed by referral to a specialist for a comprehensive neuropsychological assessment and a definitive diagnosis [3,11]. As there are no biomarkers for autism, diagnosis is based exclusively on clinical criteria, standing on personal history and clinical evaluation [12].

Identification of early markers of development in the first years of life may play a relevant clinical role in early diagnosis and allow prompt and targeted intervention [13]. One of the early symptoms that prompts caregivers of children with ASD to seek help is the presence of speech and language difficulties, commonly observed in individuals with ASD [5,14]. Emerging evidence from both retrospective and prospective studies supports the notion that behavioral signs of ASD can be identified during early infancy. It is now well-established that as many as 50% of parents can recall having concerns about their child's development dating back to the first year of life [15,16].

The aim of this study was to characterize early neurodevelopment milestones in a pre-school cohort of children with autism spectrum disorder. The authors defined a secondary aim to compare the age of acquisition of neurodevelopmental milestones between these children and the normative population by using the Haizea-Llevant development chart. It is a screening instrument that allows for the verification of the level of cognitive, social, and motor development of children from zero to five years old, offering a normal range of acquisition of some fundamental milestones during childhood [17]. A tertiary aim was to compare Griffith's Mental Development Scales-Extended Revised (GMDS-ER) psychometric evaluation results in delayed and normative acquisition of early neurodevelopmental milestones.

## Materials and methods

Study design and sample

This is a retrospective observational study of children with ASD followed at the Neurodevelopment Unit of the Centro Materno Infantil do Norte, Unidade Local de Saúde de Santo António, Porto, Portugal. Data were collected from electronic clinical records of follow-up medical appointments. This study was authorized by the hospital’s Ethics Committee; Departamento de Ensino, Formação e Investigação issued approval no. 2021.206 (167-DEFI/175-CE) to carry out the study. This study included all children with an ASD diagnosis (according to the Diagnostic and Statistical Manual of Mental Disorders, 5th Edition, Text Revision) born between 2016 and 1st of June 2022, followed at the Neurodevelopment Unit. There were no exclusion criteria.

Study variables

The following variables were collected: sex, age, age at diagnosis, referral, family history, prenatal background, and comorbidities. Early clinical manifestations of autism were also analyzed through the age of onset of the first alarm signs detected by caregivers or by healthcare or educational professionals. Regarding early neurodevelopmental milestones, we analyzed parent-reported ages at acquisition of social smile, sitting without support, walking, first words, first sentences, and urinary daytime sphincter control. Evidence of language regression [defined as consistent loss for at least three months of communicative use of at least five different words (other than "mummy" and "daddy" that were previously used for at least three months) was also assessed. Global development quotient (GDQ) and subquotients (personal/social, hearing/language, practical reasoning, locomotor, hand-eye coordination, and performance) were obtained using GMDS-ER.

Statistical analysis

Categorical variables are presented as frequencies and percentages, and continuous variables as means and standard deviations (SD). Variables with skewed distributions are presented as medians and interquartile ranges (IQR). Normal distribution was checked using the Shapiro-Wilk test of skewness and kurtosis, as appropriate. Given the Haizea-Llevant chart of psychomotor development cultural applicability and local validation, we selected six milestones: 1) “smiles at the mother's face” as social smile, 2) “sits down” as sitting, 3) “autonomous gait” as walking, 4) “daddy, mummy, with intention” as first words, 5) “two-word sentences” as first phrases and 6) “daytime sphincter control”). We chose the milestones most easily remembered by parents. Acquisition of each milestone under 95th percentile of Haizea-LLevant psychomotor development chart was defined by the authors as normative, and above or at the referred percentile was defined as delayed. Absent acquisition was also categorized as delayed. The mean or median results in GMDS-ER GDQ and subscales were compared between normative and delayed (or absent) acquisition of all milestones 1 to 6. An exception was given to locomotor subscale results that were compared between normative and delayed acquisition of the milestone of walking. Student’s t-test or Mann-Whitney for independent samples were used, as appropriate. All reported p-values are two-tailed, with p-value < 0.05 indicating statistical significance. Statistical analysis was performed using Statistical Package for the Social Sciences® software, version 29 (IBM Corp., Armonk, NY) 

## Results

Univariate analysis

Characterization of the Sample

A total of 127 children were included, of which 109 (85.8%) were male. The clinical and demographic characteristics are described in Table 1. The median age at data collection was 49 months (IQR 21), and the mean age at diagnosis was 34±10 months. Most of the children were referred from the pediatric consultation (39, 30.7%), followed by primary health care (30, 23.6%) and neonatology consultation (22, 17.3%). The remaining children came from various specialties as shown in Table 1. The main reason for referral was language impairment (53, 41.7%), followed by global developmental delay (46, 36.2%). Twenty-eight children (22.0%) already had a diagnosis of ASD at the time of the referral. The median age at the first appointment was 33 months (IQR 1).

**Table 1:** Characterization of the study population (n=127) Descriptive analysis of the sample: sex, age, age at diagnosis, referral, family history, prenatal background, and comorbidities. Categorical variables are presented as frequencies and percentages and continuous variables as means and standard deviations (SD). Variables with skewed distributions are presented as medians and interquartile ranges (IQR). * Four missing values, † 11 missing values (>5%).

Variable			Value
Children, n (%)			127 (100)
Male, n (%)			109 (85.8)
Age (months) – median (IQR)			49 (21.0)
Age at diagnosis (months) – mean±SD (min, max)			34±10 (13, 69)
Age of first alarm signs (months) – median (IQR) (min, max)			18 (11) (0, 36)
Referral			
Age of first Neurodevelopment appointment (months) – median (IQR) (min, max)			33 (1) (6, 69)
Provenance (by specialty), n (%)			
	Pediatrics		39 (30.7)
	General family medicine		30 (23.6)
	Neonatology		22 (17.3)
	Neuropediatrics		9 (7.1)
	Metabolic disorders		6 (4.7)
	Other		21 (16.5)
Motive, n (%)			
	Language disorder		53 (41.7)
	Global development delay		46 (36.2)
	Autism spectrum disorder		28 (22.0)
Family history			
Birth mother			
	Age (years) – mean±SD (min, max)		35.8±5.6 (22, 49)
	Age ≥ 40 years – n (%)		30 (23.6)
	Education – n (%)		
		Basic/secondary	* 92 (74.8)
		University	* 31(25.2)
	Unemployment – n (%)		30 (23.6)
Biological father			
	Age (years) – median (IQR) (min, max)		38 (9) (24, 66)
	Age ≥ 40 (years) – n (%)		53 (41.7)
	Education – n (%)		
		Basic/secondary	† 95 (81.9)
		University	† 21 (18.1)
	Unemployment – n (%)		7 (5.5)
Marital status			
	Married/civil union		113 (89.0)
	Divorced/single		14 (11.0)
Autism spectrum disorder			
	Yes – n (%)		20 (15.7)
Other diseases – n (%)			
	Language disorder		23 (18.1)
	Global development delay/intellectual disability		22 (17.3)
	Attention-deficit hyperactivity disorder		6 (4.7)
	Psychiatric disorders		14 (11)
	Neurological disorders		9 (7.1)
	Genetic syndromes		8 (6.3)
	Deafness		2 (1.6)
	Heart disease		2 (1.6)
	Cystic fibrosis		1 (0.8)
	Renal disease		1 (0.8)
Prenatal background			
Maternal age at gestation (years) – mean±SD (min, max)			31.6±5.6 (17, 46)
Age ≥ 40 years – n (%)			8 (6.3)
Gestational age (GA) (weeks) – median (IQR)			39 (3.0)
Prematurity (GA < 37 weeks) – n (%)			18 (14.1)
	Extreme (GA < 28 weeks)		2 (1.6)
	Moderate (GA 28 - 32 weeks)		4 (3.1)
	Late (GA 33 - 36 weeks)		12 (9.4)
Relevant prenatal history – n (%)			36 (28.3)
	Maternal diabetes (type 1, type 2 or gestational)		15 (11.8)
	Fetal growth restriction		10 (7.9)
	Drug or toxic consumption		5 (3.9)
	Maternal hypertension		3 (2.4)
	Preeclampsia		3 (2.4)
Comorbidities			
Sensory processing disorder			118 (92.9)
Sleep disorder			23 (18.1)
Disruptive, impulse control and conduct disorder			10 (7.9)
Hypotonia			9 (7.1)
Epilepsy			4 (3.1)
Other neurodevelopmental disorders			
	Language disorder		127 (100.0)
	Attention-deficit hyperactivity disorder		7 (5.5)
	Motor developmental coordination disorder		4 (3.1)
	Tics disorder		4 (3.1)

Approximately a quarter of the mothers (23.6%) had ≥ 40 years, 74,8% had basic or secondary education and 25.2% went to the university (vs. 18.1% of fathers). Maternal unemployment was higher than paternal (23.6% vs. 5.5%). The majority of parents were married or in a civil union (89%). Regarding family medical history (1st or 2nd degree relatives), 20 (15.7%) had at least one close relative with ASD. Approximately half (55.1%) reported other family disorders (neurodevelopmental, psychiatric and neurological disorders). Noting that in 19 (15%), more than one condition was reported. In the prenatal background, the mean maternal age was 31.6 ± 5.6 years. The median gestational age was 39 weeks (IQR 3), and 14.1% of infants were born preterm. Overall, 27.6% had relevant prenatal history (6.3% with more than one reported condition).

Among these children with ASD, the most common co-morbidity was sensory processing disorder in 118 (92.9%), of these 89 (71.8%) vestibular, 85 (68.5%) tactile, 69 (55.6%) auditory, 65 (52.4%) proprioceptive, 85 (44.4%) taste, 38 (30.6%) visual, and three (2.4%) olfactory. Other comorbidities included sleep disorders, hypotonia, epilepsy, as well as psychiatric and neurodevelopmental disorders. All children had a language disorder.

Neurodevelopmental Milestones

**Figure 1:**
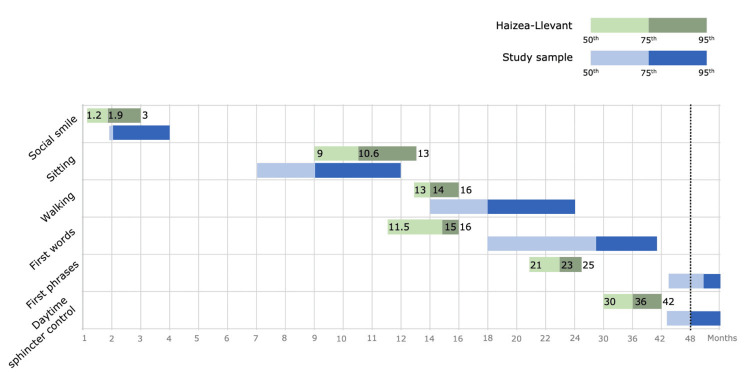
Haizea-LLevant and study sample (n=127) 50th, 75th and 95th percentiles of milestones ages of acquisition Representation of our study selected milestones corresponding to Haizea-Llevant chart of psychomotor development. For each neurodevelopmental milestone presented on the left are exposed by the blue bars the 50th, 75th and 95th percentiles of the acquisition age (by months). Above them, the green bars represent the respective percentiles defined by Haizea-LLevant psychomotor development chart.

For each neurodevelopmental milestone, the 50th, 75th, and 95th percentiles of the acquisition age were registered and presented in Figure 1 (blue bars). Above them are the respective percentiles defined by Haizea-LLevant psychomotor development chart (green bars). 

'Social smile' was acquired at a median age of two months (IQR 0) (50th percentile 2, 75th percentile 2, 95th percentile 4). 101 (79.5%) acquired it at normative age, and 26 (20.5%) had delayed acquisition (later than three months). 'Sitting' was acquired at a median age of seven months (IQR 3) (50th percentile 7, 75th percentile 9, 95th percentile 12); 115 (97.5%) sat within the expected age, with 2.5% (n=3) of delay. 'Walking' was acquired at a median age of 14 months (IQR 6) (50th percentile 14, 75th percentile 18, 95th percentile 24). Normative acquisition occurred in 78 (61.4%) and delayed acquisition in 49 (38.6%), noting that two children (1.6%) weren’t able to walk. 'First words' were said at a median age of 18 months (IQR 16) (50th percentile 18, 75th percentile 28, 95th percentile 41.4). There were more children delayed than normative (79, 62.6%) vs 47 (37.3%) and 14 (11.1%) haven’t said any word. 'First phrases'were acquired at a mean age of 41.8±10.9 months (50th percentile 44, 75th percentile 51.3, 95th percentile 59.3). The majority of children (122, 96.8%) showed delayed acquisition, with this milestone having the highest percentage of non-acquisition (92, 73%). 'Daytime sphincter control' was acquired at a median age of 43 months (IQR 12) (50th percentile 43, 75th percentile 48, 95th percentile 66.4). One hundred and four (85.2%) children had delayed acquisition, 79 (64.8%) had no acquisition, and only 18 (14.8%) acquired it at a normative age. Fourteen (11%) of the children had regression, occurring at a mean age of 20.3±3.9 months. All the results are presented in Table 2.

**Table 2:** Neurodevelopmental milestones acquisition (total n=127, GMDS-ER performed n=79) Presentation of the acquisition age for the six milestones, indicating the number of children who achieved them at a normative age, experienced delayed acquisition, or did not acquire them. Acquisition of each milestone under the 95th percentile of Haizea-LLevant psychomotor development chart was defined as normative, and above or at the referred percentile was defined as delayed. Absent acquisition was also categorized as delayed. GMDS-ER: Griffiths Mental Development Scales-Extended Revised. ‡ Nine missing values (>5%), § One missing value, ¶ Five missing values, ** Eight missing values (>5%), †† Three missing values.

Neurodevelopmental milestones				Total (n=127)	GMDS-ER (n=79)
Social smile					
	Age (months) – median (IQR)			2 (0)	2 (0)
	Acquisition – n (%)				
		Normative		101 (79.5)	64 (81.0)
		Delayed		26 (20.5)	15 (19.0)
Sitting					
	Age (months) – median (IQR)			7 (3.0)	7 (3.0)
	Acquisition – n (%)				
		Normative		‡ 115 (97.5)	** 71 (100.0)
		Delayed		‡ 3 (2.5)	0 (0)
Walking					
	Age (months) – median (IQR)			14 (6.0)	14 (5.0)
	Acquisition – n (%)				
		Normative		78 (61.4)	49 (62.0)
		Delayed		49 (38.6)	30 (38.0)
			Absent	2 (1.6)	0 (0)
First words					
	Age (months) – median (IQR)			18 (16.0)	18 (16.0)
	Acquisition – n (%)				
		Normative		§ 47 (37.3)	§ 28 (35.9)
		Delayed		§ 79 (62.6)	§ 50 (64.1)
			Absent	14 (11.1)	8 (10.3)
First phrases					
	Age (months) – mean±SD			41.8±10.9	42.9±10.7
	Acquisition – n (%)				
		Normative		§ 4 (3.2)	** 2 (2.6)
		Delayed		§ 122 (96.8)	** 76 (97.4)
			Absent	92 (73.0)	55 (70.5)
Daytime sphincter control					
	Age (months) – median (IQR)			43 (12.0)	44 (11.0)
	Acquisition – n (%)				
		Normative		¶ 18 (14.8)	†† 12 (15.8)
		Delayed		¶ 104 (85.2)	†† 64 (84.2)
			Absent	79 (64.8)	42 (55.3)
Language regression					
	Yes – n (%)			14 (11.0)	8 (10.1)
	Age (months) – mean±SD			20.3±3.9	19.5±4.2

Psychometric Evaluation

Psychometric evaluation was performed using GMDS-ER, whose application was possible in 79 (62.2%) children, median age 36 (16) months. Mean GDQ was 61.8±12.7. Results in GMDS-ER subscales were the following: personal/social median 58 (IQR 19.2), hearing/language mean 45±14.5, locomotor median 80 (IQR 18), hand-eye coordination mean 61.9±14.9, and performance mean 69±19.2. The practical reasoning subscale was excluded as it was only possible to apply it to a small number of children.

Bivariate analysis

Median GDQ in children with delayed daytime sphincter acquisition was significantly lower than in children without delayed acquisition of this milestone (60.2 (11.8) vs 76.2 (17.8), p=0.002). Median personal/social results were significantly lower in children with delayed social smile than in children without delayed social smile (51.1 (13.4) vs 60 (18.8), p=0.03). This GMDS-ER subscale had also lower results comparing delayed and normative acquisition of first words” (56.7 (14.4) vs 64 (23.8) vs, p=0.014), first sentences (58 (17.1) vs 80.5 (19.2), p=0.023), and daytime sphincter control (56.7 (14) vs 70 (16.3), p<0.01). Median results of the hearing/language subscale in children with delayed daytime sphincter acquisition were significantly lower than in children without delayed acquisition of this milestone (42 (16.4) 51.7 (26.3), p=0.048). Hand-eye coordination subscale results did not have statistically significant differences between normative and delayed acquisition of all milestones. Median results of performance subscale in children with delayed daytime sphincter acquisition were significantly lower than in children without delayed acquisition of this milestone (69.3 (18.9) vs 78 (26.8), p=0.011). Mean results on this subscale in children with delayed walking were also significantly lower than in children with normative acquisition of this milestone (62.8±18.9 vs 72.8±18.6, p=0.025). All results are presented in Table 3.

**Table 3:** GMDS-ER results for milestones normative and delayed acquisition (n=79) Means or medians results in Griffiths Mental Development Scales-Extended Revised (GMDS-ER) GDQ and subscales (personal/social, hearing/language, hand-eye coordination, performance) comparison between normative and delayed (or absent) acquisition of all milestones ("sitting" was excluded as there was no delayed acquisition of this milestone). The locomotor subscale results were compared between normative and delayed acquisition of the milestone walking. Student’s t-test or Mann-Whitney for independent samples were used, as appropriate. All reported p-values are two-tailed, with p-value < 0.05 indicating statistical significance.

		Normative acquisition	Delayed acquisition	p value
Global Development Quotient (GDQ)				
	Social smile – mean±SD	62.6±12.4	58.1±13.8	0.212
	Walking – mean±SD	63.8±11.8	58.4±13.6	0.066
	First words – mean±SD	64.4±15.3	60.1±10.8	0.148
	First sentences – median (IQR)	74.2 (16.2)	61.2 (14.6)	0.118
	Daytime sphincter control – median (IQR)	76.2 (17.8)	60.1 (11.8)	0.002
Personal/Social				
	Social smile – median (IQR)	60 (18.8)	51.1 (13.4)	0.03
	Walking – median (IQR)	58 (19)	57.7 (17.7)	0.396
	First words – median (IQR)	64 (23.8)	56.7 (14.4)	0.014
	First sentences – median (IQR)	80.5 (19.2)	58 (17.1)	0.023
	Daytime sphincter control – median (IQR)	70 (16.3)	56.7 (14)	< 0.01
Hearing/Langauge				
	Social smile – mean±SD	46.3±14.5	39.2±13.2	0.09
	Walking – median (IQR)	43.8 (15)	41 (20.9)	0.874
	First words – mean±SD	49.1±16.9	42.7±15.6	0.064
	First sentences – median (IQR)	59.6 (18.1)	42.5 (16.8)	0.248
	Daytime sphincter control – median (IQR)	51.7 (26.3)	42 (16.4)	0.048
Locomotor				
	Walking – mean±SD	80.9±14.5	74.6±17	0.083
Hand-eye coordination				
	Social smile – mean±SD	62.7±14.5	58.5±16.7	0.324
	Walking – mean±SD	63.3±14.5	59.7±15.7	0.311
	First words – mean±SD	62.2±18	62±13.3	0.956
	First sentences – median (IQR)	62.2 (16.7)	64 (15.9)	0.975
	Daytime sphincter control – mean±SD	66.7±20	61.1±13.4	0.371
Performance				
	Social smile – median (IQR)	72 (21.5)	64.9 (21)	0.5
	Walking – mean±SD	72.8±18.6	62.8±18.9	0.025
	First words – median (IQR)	73.5 (26.6)	70.1 (17.4)	0.2
	First sentences – median (IQR)	88.5 (21.2)	68.3 (20.5)	0.091
	Daytime sphincter control – median (IQR)	78 (26.8)	69.3 (18.9)	0.011

## Discussion

The mean age at the diagnosis of ASD of our sample was 34 months, which is in line with what is described in other studies, indicating that the typical age of diagnosis is around three or four years old. However, it has been observed that children from low socioeconomic backgrounds or without a family history of ASD may receive a diagnosis at a later age [16]. Language delay was the main reason for referral to the Neurodevelopment appointment, highlighting the importance of considering autism as a possible condition for a child presenting with this impairment.

In our study, family history of ASD as well as neurodevelopmental, neurological, and psychiatric disorders, has a high representation as described in other studies. Co-occurring conditions are common in children with ASD and may have a great effect on the child and family function and clinical management. As in our study, other authors report comorbid conditions like ADHD, motor developmental coordination disorder, sleep disorder, sensory processing disorder, and psychiatric disorders, namely anxiety or oppositional defiant disorders [7,18-20].

Regression had occurred in 14 (11%) of the children, with a mean age of 20.3±3.9. Some studies support our results showing that regression is present in 30% occurring before three years, with peak age 13-18 months [7,14]. Identification of early markers of development in the first years of life may play a relevant clinical role in early diagnosis and allow estimation in terms of clinical severity and individualization of child and family needs. In our study, during the first year of life, motor milestones were achieved within the expected timeframe, except for independent walking, which was delayed when compared to those with the same age and a typical development in the Haizea-LLevant development chart. The onset of this milestone occurred at a median age of 14 months in our group of patients with autism. In fact, some studies show that the onset of motor skills has been proven to be affected in patients with autism [12,21]. In our study, the delayed onset of walking was associated with lower non-verbal intellectual skills, shown by the association of lower results on the performance subscale, also found in other studies [12,21].

About the acquisition of first words, especially first phrases, percentile bars are completely apart from Haizea-LLevant, showing that these milestones are acquired later in autism. While in typically developing children, the last two milestones are acquired before 42 months, in our sample, they have not yet been acquired at 49 months. We can consider that they may still be acquired, but according to Hong-Hua Li et al., children with ASD have rapid language development between 24 and 48 months, suggesting that the level of language development at 48 months could serve as an indicator of the language prognosis [22]. It is clear that language plays a significant role in various aspects of everyday life, affecting outcomes such as academic success, experiences of bullying, and interpersonal relationships, so it is crucial to address and understand it to support individuals in navigating diverse social, academic, and personal contexts effectively [23].

The delay in acquiring daytime urinary sphincter control was related to many subscales assessed in the GMDS-ER evaluation, namely GDQ, personal/social, hearing/language, and performance subscales. This indicates that it could be an important milestone to be aware of due to its association with lower intellectual skills [21,24,25]. Other studies found a higher prevalence of incontinence in children with ASD when compared to typically developing children [26,27]. The personal/social subscale evaluates a child's skills in daily activities and their ability to engage with other children, encompassing items such as "smiling," "tracking moving people with their eyes" and "clapping in imitation" to assess social learning and the acquisition of social behaviors. Social experiences play a vital role in developing cognitive processes that enable individuals to interact effectively with their surroundings [5,10]. This dimension was the one that showed the strongest correlation with early neurodevelopmental milestones.

Children who experienced delays in acquiring social smile, first words, first phrases, and sphincter control had significantly lower outcomes compared to those who achieved these milestones within the expected age. Early identification of delays in these milestones allows for prompt intervention services that greatly enhance the prognosis for children, as opposed to delaying behavior management and education [14]. There are limitations to this study that should be noted. First, the study design is retrospective, which introduces the possibility of selection bias and recall bias. Second, the reduced sample size is also a limitation to drawing conclusions that can be generalized. Third, the absence of GMDS-ER evaluation of all children. Fourth, the sample age does not allow for a definitive characterization of individuals' competencies. Lastly, this is a biased sample of children followed in the Neurodevelopment unit.

## Conclusions

Our study enhances the relevance of careful clinical records of the onset age of psychomotor developmental milestones as crucial elements for the natural history of autism, as these are associated with clinical presentation of autism, as well as with cognitive and adaptive functioning. Furthermore, the identification of early developmental milestones in children with ASD is crucial for early diagnosis and intervention. Our study evaluated the developmental trajectories of a cohort of children with ASD in order to identify potential markers of early identification. In fact, by reviewing information on developmental milestones, including language and communication, motor skills, and social communication, this study can contribute to informing the development of more effective screening and intervention strategies, which may improve long-term outcomes for children with ASD.
